# Microbial Profiling and Biosafety Assessment of a *Sargassum-*Based Liquid Biofertilizer Using 16S rRNA Metagenomics

**DOI:** 10.1155/ijm/3219583

**Published:** 2026-04-24

**Authors:** Yaset Rodríguez-Rodríguez, Ashley Marie Mejía Disla, Máximo Elías Reynoso Ortega, Gustavo Gandini, Pamela Tejada-Tejada, Miguel Ángel Guevara, Edian F. Franco, Carlos Willian Dias Dantas, Rommel T. Ramos, Ulises Javier Jáuregui-Haza

**Affiliations:** ^1^ Área de Ciencias Básicas y Ambientales, Instituto Tecnológico de Santo Domingo (INTEC), Santo Domingo, Dominican Republic, intec.edu.do; ^2^ Asociación BANELINO, Mao, Dominican Republic; ^3^ Instituto Superior de Formación Docente Salomé Ureña (ISFODOSU), Santo Domingo, Dominican Republic; ^4^ Genomics and Bioinformatics Laboratory, Department of Research and Scientific Production, Universidad Tecnológica de Santiago (UTESA), Santiago de los Caballeros, Dominican Republic; ^5^ Department of Research and Innovation, Instituto de Innovacción en Biotecnología e Industria (IIBI), Santo Domingo, Dominican Republic; ^6^ Department of Biochemistry and Immunology, Institute of Biological Sciences, Federal University of Minas Gerais, Belo Horizonte, Minas Gerais, Brazil, ufmg.br; ^7^ Laboratory of Bioinformatics and Genomics of Microorganisms, Institute of Biological Sciences, Federal University of Pará, Belém, Pará, Brazil, ufpa.br

**Keywords:** 16S rRNA, liquid biofertilizer, metagenomics, microbial diversity, *Sargassum*, sustainable agriculture

## Abstract

*Sargassum* seaweed is increasingly abundant in the Caribbean, creating ecological disruption but also providing biomass for agricultural inputs. This study compares the microbial diversity and safety of a *Sargassum*‐based liquid biofertilizer (SBLB‐INTEC) with those of a conventional product (LB‐BANELINO) using 16S rRNA amplicon sequencing, rather than culture‐dependent methods. Both formulations contained key nutrients (K, Ca, and Mg) and low levels of heavy metals. They harbored dense but relatively simple bacterial communities dominated by Firmicutes, particularly Bacilli, with Proteobacteria and other phyla at lower abundances. *Staphylococcus* (Staphylococcaceae) was highly abundant in both products, while SBLB‐INTEC showed a somewhat more balanced community, including *Delftia* and other Comamonadaceae. Shannon diversity tended to be higher in SBLB‐INTEC, but differences in alpha‐ and beta‐diversity between formulations were not statistically significant. Because 16S data cannot distinguish viable from nonviable cells or resolve strain‐level pathogenicity, these results do not prove the absence of pathogens; instead, they provide a genus‐level baseline to guide targeted culture, qPCR, and functional assays. Overall, the combination of a favorable chemical profile and microbial groups commonly associated with nutrient cycling and plant‐associated functions suggests that SBLB‐INTEC could become a valuable component of integrated nutrient management in tropical agriculture, offering hope for a more sustainable future pending confirmatory plant‐response and biosafety studies. We recommend integrating these microbial data into a national biofertilizer monitoring framework, combining metagenomic surveys with targeted qPCR and resistance gene screening.

**Highlights**
• First 16S rRNA amplicon profiling of the microbial community of a *Sargassum*‐based liquid biofertilizer produced in the Dominican Republic.
• The biofertilizers contained dense but relatively simple bacterial communities dominated by Firmicutes*/*Bacilli*.*
• 16S rRNA sequencing provided a genus‐level map of potentially beneficial and opportunistic microbial groups.
• Microbial diversity supports the safety and sustainable use of agriculture.

## 1. Introduction

In recent years, the Caribbean has experienced unprecedented accumulations of *Sargassum* seaweed, disrupting coastal economies and ecosystems [1–3]. Beyond its negative impacts, such as beach fouling, habitat loss, and economic burdens, *Sargassum* can be a valuable biomass for circular economic solutions, including the production of liquid biofertilizers [4, 5]. Prior investigations demonstrate that *Sargassum* is rich in nitrogen, potassium, and micronutrients, which are beneficial for plant growth [6, 7]. However, the same macroalgae can accumulate heavy metals in the marine environment, raising concerns about food safety and the environment [2, 8].

Recent research on *Sargassum*‐derived biofertilizers has primarily focused on heavy metal and nutrient concentrations and agronomic efficacy [9]. In our previous study, we assessed their microbiological quality using conventional selective culture techniques, which, while valuable, may not detect the full diversity of microorganisms, particularly noncultivable or low‐abundance taxa [10]. However, limited attention has been paid to their microbiological composition and its implications for safety and effectiveness. Understanding the microbial communities in biofertilizers is crucial because beneficial microbes can improve soil health and plant growth, while potential pathogens could endanger agricultural systems. This study aims to fill this gap by examining the microbial diversity and potential biosecurity risks associated with *Sargassum*‐based liquid biofertilizers (SBLB‐INTEC).

To overcome the limitations of culture‐dependent methods, metagenomic sequencing has emerged as a robust tool to characterize microbial diversity with greater sensitivity and resolution [11]. Genomic analysis of biofertilizer samples is becoming essential for accurately describing the microbial communities present in these bioproducts [12]. The widespread use of DNA sequencing over the past few decades has played a pivotal role in accurately understanding microbial ecology and detecting specific functional groups within microbial populations [13]. DNA barcoding utilizes standardized species‐specific genomic regions (DNA barcodes) to generate vast DNA libraries, aiding in identifying unknown specimens [14]. This methodology is crucial, particularly for bacteria with unusual phenotypic profiles, slow‐growing or uncultivable bacteria, and culture‐negative infections [15].

Regarding biofertilizers, metagenomic techniques like 16S rRNA sequencing allow for the identification of microbial communities involved in nutrient cycling and promoting plant growth, while also confirming the absence of potentially harmful microorganisms. Therefore, this method can offer deeper understanding of both functional capabilities and biosecurity concerns.

The objective of this study was to characterize the bacterial communities in a SBLB‐INTEC produced in the Dominican Republic and in a conventional biofertilizer used as a reference, using 16S rRNA amplicon sequencing as an initial culture‐independent survey of microbial diversity. By combining these data with previously reported chemical and agronomic information [9, 10, 16], we aim to provide an initial, evidence‐based assessment of the potential agronomic value and biosecurity considerations associated with the use of SBLB‐INTEC in Caribbean agriculture. A preprint of this work has been made available elsewhere [16].

## 2. Materials and Methods

### 2.1. Biofertilizer Production

The experimental phase took place from May to December 2024. During this time, two liquid biofertilizers were produced at the BANELINO Association’s facilities in Montecristi, Dominican Republic. The Association consists of small‐scale banana farmers committed to sustainable, organic farming.

SBLB‐INTEC was created using *Sargassum* collected in Punta Cana, Dominican Republic, combined with molasses, baker’s yeast (*Saccharomyces cerevisiae*), yogurt, milk, and water from the Yaque del Norte River. The mixture went through anaerobic fermentation for about 30 days in a 1000 L static reactor.

LB‐BANELINO (control) was prepared with *Sargassum*, using molasses and a “mother culture” containing forest mulch, rice bran, and grass, fermented under similar anaerobic conditions.

Physicochemical characterization and elemental content of both biofertilizers were determined using standard protocols previously used in research [10]. Table 1 shows the concentrations of alkali/alkaline‐earth metals, selected heavy metals, basic biomolecules, and physicochemical parameters for both biofertilizers.

**TABLE 1:** Physicochemical, basic biomolecules, and elemental profile of the SBLB‐INTEC and LB‐BANELINO biofertilizers.

Parameter	Unit	SBLB‐INTEC	LB‐BANELINO	Reference range
*Alkali and alkaline earth metals*				
Potassium (K)	mg/kg	5996.00	2786.00	3120.00^∗^
Calcium (Ca)	mg/kg	2042.00	730.00	—
Magnesium (Mg)	mg/kg	762.00	295.00	628.00^∗^
Sodium (Na)	mg/kg	599.00	204.00	948.00^∗^

*Heavy metals*				
Lead (Pb)	mg/kg	< 1.50	< 1.50	100–1000^∗∗^
Cadmium (Cd)	mg/kg	< 0.20	< 0.20	0.08–0.20^∗^, 1–37^∗∗^
Arsenic (As)	mg/kg	< 0.50	< 0.50	15–50^∗∗^
Copper (Cu)	mg/kg	4.00	8.00	0.10–0.90^∗^, 60–200^∗∗^
Iron (Fe)	mg/kg	25.40	104.00	1.60–55^∗^
Zinc (Zn)	mg/kg	< 3.00	12.80	0.15–7.5^∗^, 70–600^∗∗^

*Biomolecules and physicochemical parameters*				
Total carbohydrates	g/100 g	0.73	< 0.20	—
Total proteins	g/100 g	0.2	0.27	—
Total lipids	g/100 g	1	1	—
pH	—	4.9	3.7	—
Electrical conductivity	μS/cm	3654	1404	—
Salinity	ppt	15.9	6.6	—

^∗^Reference values found in other liquid biofertilizer [17–23].

^∗∗^Values permissible limits in agricultural soils [24–37].

### 2.2. Microbiological Composition Analysis

#### 2.2.1. DNA Extraction

Biofertilizer samples were prefiltered using a vacuum‐assisted system with a 1.6 μm cellulose membrane to remove coarse particles, followed by a 0.2 μm membrane to retain microbes. Membranes were stored in Tris‐EDTA (TE) buffer at −20°C until processing. Metagenomic DNA was extracted using the DNeasy PowerWater Kit (Qiagen, Germany) according to the manufacturer’s instructions, after thawing at room temperature.

#### 2.2.2. 16S rRNA Amplicon Sequencing

DNA concentration and purity were first evaluated using a NanoDrop ND‐1000 spectrophotometer (NanoDrop Technologies), and DNA integrity was verified by agarose gel electrophoresis. The hypervariable V3–V4 region of the bacterial 16S rRNA gene was amplified using the universal primer pair 341F (5′‐CCTAYGGGRBGCASCAG‐3′) and 806R (5′‐GGACTACNNGGGTATCTAAT‐3′), following Novogene’s standard 16S amplicon metagenomic workflow for Illumina NovaSeq platforms (paired‐end 2 × 250 bp).

PCR products were purified and used for library construction according to the provider’s protocols. Library quantity was measured with a Qubit 2.0 fluorometer (Thermo Fisher Scientific, Waltham, MA, USA), and fragment size distribution and integrity were assessed with an Agilent 2100 Bioanalyzer (Agilent Technologies, Santa Clara, CA, USA). High‐quality libraries were sequenced on an Illumina NovaSeq 6000 system (2 × 250 bp), generating V3–V4 16S rRNA amplicon datasets for downstream community and diversity analyses. The raw 16S rRNA amplicon sequencing data generated in this study have been deposited in the NCBI Sequence Read Archive (SRA) under BioProject accession number PRJNA1237349, entitled “Project for the metagenomic analysis of Dominican biofertilizers.” The BioProject includes six BioSamples and six corresponding SRA records. Associated sample metadata and sequencing quality summaries are provided as Supporting Table S4A.

#### 2.2.3. Bioinformatic Processing and ASV Inference

Sequencing was performed in triplicate for each group: SBLB‐INTEC and LB‐BANELINO. Sequencing was performed on six samples corresponding to two biofertilizer formulations: LB‐BANELINO (BB1, BB2, and BB3) and SBLB‐INTEC (BSI1, BSI2, and BSI3). A complete metadata file, including sample identifiers, biofertilizer type, replicate number, production batch, and sampling site, was used for downstream bioinformatic and statistical analyses and is provided as Supporting Table S4A.

Quality filtering and read preprocessing were performed following the standard Novogene amplicon pipeline [38, 39]. Adapter, barcode, and primer sequences were removed, paired‐end reads were merged, and low‐quality reads (Phred score < 20) were discarded. After quality filtering and chimera removal, an average of approximately 97% of reads per sample were retained for downstream analyses.

Amplicon sequences were processed on the Galaxy Europe platform using the QIIME 2 amplicon distribution (Version 2025.10), which offers a fully reproducible environment for microbiome analysis [40]. Paired‐end FASTQ files for each biofertilizer sample were imported, and initial quality control was conducted with FastQC to examine per‐base quality scores, GC content, and sequence length distributions [41]. Individual FastQC reports were aggregated with MultiQC to obtain a global summary of read quality and potential artifacts across all libraries [42] (Table S6A). These summaries guided the choice of trimming and truncation parameters for denoising.

Sample information (biofertilizer type, sampling site, and replicate) was stored in tab‐separated metadata files in accordance with QIIME 2 conventions. A PairedEndFastqManifestPhred33V2 manifest file was created to link each sample ID to the absolute paths of its forward and reverse reads. Paired‐end reads were imported into QIIME 2 via the Galaxy “qiime2 tools import‐fastq” interface, using the manifest file and the PairedEndFastqManifestPhred33V2 format. The resulting demultiplexed artifact was summarized with qiime demux summarize to obtain per‐base quality plots and per‐sample sequence counts.

Error correction, chimera removal, and inference of amplicon sequence variants (ASVs) were performed with the DADA2 plugin (qiime dada2 denoise‐paired), a model‐based approach that resolves exact sequence variants from Illumina amplicon data [43]. Forward and reverse reads were processed with the following key parameters: trunc_len_f = 250, trunc_len_r = 250, trim_left_f = 0, trim_left_r = 0, max_ee_f = 2, max_ee_r = 2, trunc_q = 2, min_overlap = 12, max_merge_mismatch = 0, chimera removal by chimera_method = consensus, and *n*_reads_learn = 1,000,000. DADA2 generated a feature table of ASV counts, a set of representative ASV sequences, and detailed denoising statistics for each sample. These outputs were examined with qiime feature‐table summarize and qiime feature‐table tabulate‐seqs to verify sequence length distributions, per‐sample sequencing depth, and the number of ASVs retained after quality control.

Taxonomic assignment was performed using the QIIME 2 feature‐classifier plugin and a naïve Bayes classifier trained on the SILVA 138 reference database, which was trimmed to the V3–V4 region targeted in this study. The classifier (qiime feature‐classifier classify‐sklearn) was applied to the representative ASV sequences, yielding a taxonomy table with annotations ranging from phylum to species, where possible. Relative abundance barplots at the phylum, class, order, family, and genus levels were generated with qiime taxa barplot, using the ASV table, taxonomy assignments, and the final metadata file to inspect community composition and evaluate the consistency of replicates within each biofertilizer formulation.

For downstream statistical analyses in Python (Google Colab), the ASV abundance table was exported from Galaxy in BIOM format (feature‐table.biom) using qiime tools export, and the taxonomy table was exported as a tab‐separated file (taxonomy.tsv). The resulting ASV count matrix, relative abundance table, and taxonomy table are provided as Supporting Tables S1, S2, and S3, respectively.

#### 2.2.4. Phylogenetic Reconstruction, Diversity Metrics, and Statistics

To compute phylogeny‐based diversity metrics, a rooted ASV phylogenetic tree was constructed following the standard QIIME 2 phylogeny workflow. Representative ASV sequences were aligned using MAFFT, a multiple‐sequence alignment algorithm optimized for large datasets (qiime alignment mafft) [44]. The alignment was masked to remove highly variable positions using Qiime’s alignment mask. A maximum‐likelihood phylogenetic tree was then inferred with FastTree (qiime phylogeny fasttree), which efficiently estimates approximately maximum‐likelihood trees for large alignments [45], and was midpoint‐rooted with qiime phylogeny midpoint‐root.

Phylogenetic and nonphylogenetic diversity metrics were calculated using the QIIME 2 diversity core‐metrics‐phylogenetic pipeline, combining the rooted tree, the final feature table, and the updated metadata. Samples were rarefied to a sequencing depth close to the minimum postfiltering read count (∼1.6 × 10^5^ sequences per sample), ensuring comparable information content across libraries. The pipeline generated a rarefied ASV table, vectors for Faith’s phylogenetic diversity (PD), observed ASVs, Shannon diversity, and Pielou’s evenness, as well as distance matrices for unweighted and weighted UniFrac, Jaccard, and Bray–Curtis, together with their corresponding PCoA ordinations and Emperor visualizations. Per‐sample alpha diversity values and between‐formulation statistics are summarized in Supporting Tables S9 and S10. In contrast, the Bray–Curtis distance matrix, ordination coordinates, and PERMANOVA results are provided in Supporting Tables S11, S12, and S13, respectively.

For figure generation and additional hypothesis‐driven statistics, the Bray–Curtis PCoA results were exported using qiime tools export and imported into Python. Shannon diversity indices were compared between biofertilizer types using Mann–Whitney *U* tests, and a PERMANOVA was performed on the Bray–Curtis distance matrix using 999 permutations [46] to test for differences in community composition between formulations. High‐resolution barplots for the top 10 taxa at different taxonomic levels, alpha‐diversity boxplots, and PCoA ordination plots were generated from the exported ASV, taxonomy, metadata, and ordination tables. Sequencing read counts at each processing step, including raw reads, reads retained after quality trimming and merging, reads remaining after chimera removal, and final reads used for taxonomic assignment, are summarized in Supporting Table S5A.

## 3. Results and Discussion

### 3.1. Microbial Composition of Biofertilizers

Sequencing of the 16S rRNA gene (V3–V4 region) and processing with QIIME 2/DADA2 generated a high‐resolution table of ASVs. After quality control, filtering, and chimera removal, 1.6 × 10^5^–1.8 × 10^5^ reads per sample were obtained, with an average of approximately 1.7 × 10^5^ reads per sample (Supporting Table S1). Alpha diversity indices (Chao1, Shannon, and Simpson) for each sample are reported in Supporting Table S9, and summary statistics for comparisons between formulations are shown in Supporting Table S10; no additional rarefaction was applied.

ASVs were taxonomically assigned using a Naive Bayes classifier trained with SILVA 138 trimmed to the V3–V4 region. The comprehensive presentation of the relative abundance data at the phylum, class, order, family, and genus levels for each replicate in Supporting Tables S4, S5, S6, and S7, as well as in Supporting Table S8 for the main genera.

Rarefaction analyses based on observed ASVs and Shannon diversity are shown in Supporting Figure S1A. In all samples, rarefaction curves reached a clear plateau at sequencing depths well below the maximum number of reads obtained, indicating that the sequencing effort was sufficient to capture most of the bacterial richness and community diversity present in each biofertilizer formulation.

Alpha diversity analyses based on observed ASVs and Faith’s PD indicated comparable levels of within‐sample diversity across biofertilizer formulations. Observed ASVs reflected similar richness patterns among samples, while Faith’s PD values suggested that the evolutionary breadth of bacterial communities was broadly consistent between LB‐BANELINO and SBLB‐INTEC.

#### 3.1.1. Community Structure at the Phylum and Class Levels

**FIGURE 1:**
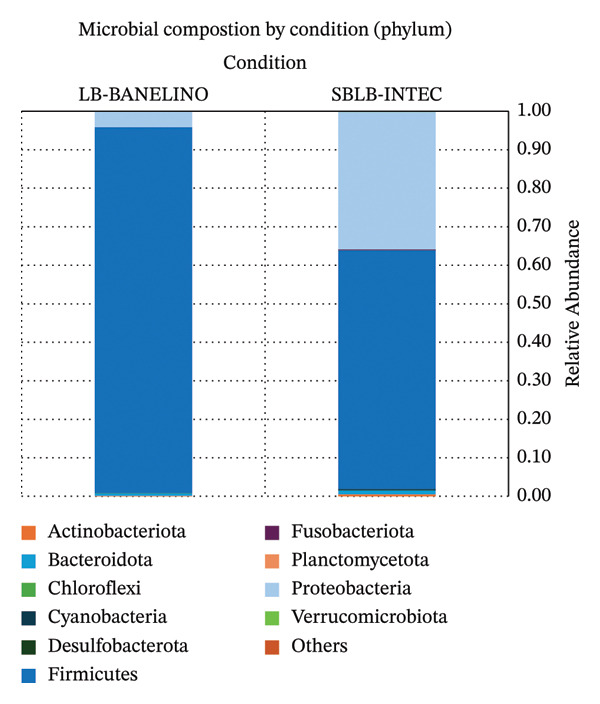
Relative abundance of bacterial phyla in SBLB‐INTEC and LB‐BANELINO biofertilizers. Stacked bar plots showing the relative abundance (%) of the main bacterial phyla in each replicate of SBLB‐INTEC (SBLB_rep1–3) and LB‐BANELINO (BANELINO_rep1–3), based on 16S rRNA (V3–V4) amplicon sequencing.

In both biofertilizers, the bacterial community was strongly dominated by Firmicutes, followed by Proteobacteria and, to a lesser extent, Bacteroidota and Actinobacteriota (Figure 1; Supporting Table S4, relative abundance of phyla by sample).

In LB‐BANELINO, Firmicutes accounted for an average of 95% of the reads, while Proteobacteria accounted for around 4%. In SBLB‐INTEC, Firmicutes remained the dominant phylum, but with a lower relative proportion (61%), accompanied by a marked increase in Proteobacteria (36%). The other phyla were below 1% in both formulations.

**FIGURE 2:**
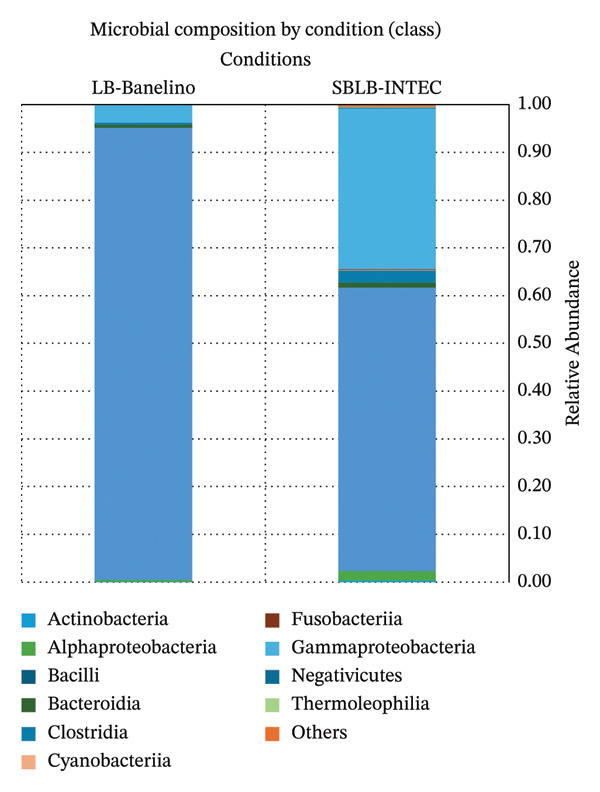
Relative abundance of bacterial classes in SBLB‐INTEC and LB‐BANELINO biofertilizers. Stacked barplots of the relative abundance (%) of bacterial classes in each replicate of SBLB‐INTEC and LB‐BANELINO.

At the class level, the community was dominated by Bacilli, consistent with the predominance of Firmicutes Figure 2; Supporting Table S5, relative abundance of bacterial classes by sample). In LB‐BANELINO, Bacilli accounted for approximately 94% of the relative abundance. In comparison, in SBLB‐INTEC, it decreased to 58%, offset by an increase in Gammaproteobacteria (33%) and, to a lesser extent, Clostridia, Alphaproteobacteria, and Bacteroidia.

This pattern is consistent with the widely documented role of Firmicutes, particularly Bacilli, in the degradation of organic matter, the mineralization of nutrients, and the synthesis of bioactive molecules such as siderophores and antimicrobial compounds. These compounds can contribute to the suppression of soil pathogens and stress tolerance in plants, as demonstrated by [47, 48]. The significant presence of Bacilli in both products suggests a potential to support soil fertility and resilience in agricultural systems such as small‐ and medium‐scale banana plantations in the Dominican Republic, where the use of local biofertilizers can reduce dependence on synthetic inputs.

Proteobacteria and the Gamma*-* and Alphaproteobacteria classes include numerous taxa associated with nitrogen fixation, denitrification, and phosphorus solubilization [49, 51]. The greater relative contribution of Gammaproteobacteria in SBLB‐INTEC compared to LB‐BANELINO suggests a potentially more diverse community in terms of nutrient transformation functions. However, this interpretation should be considered a taxonomic inference rather than direct evidence of functional genes.

#### 3.1.2. Composition at the Order, Family, and Genus Level

**FIGURE 3:**
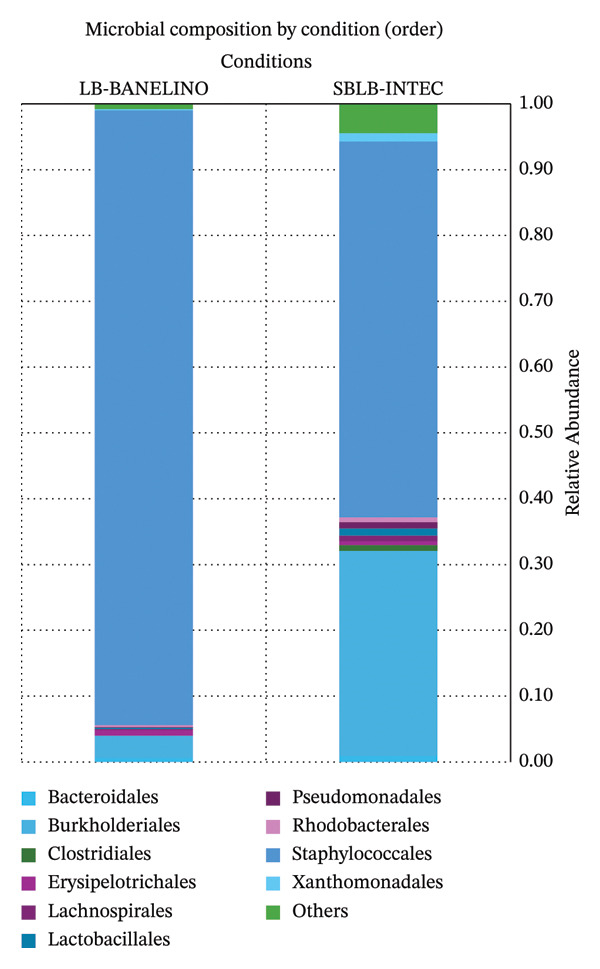
Relative abundance of bacterial orders in SBLB‐INTEC and LB‐BANELINO.

**FIGURE 4:**
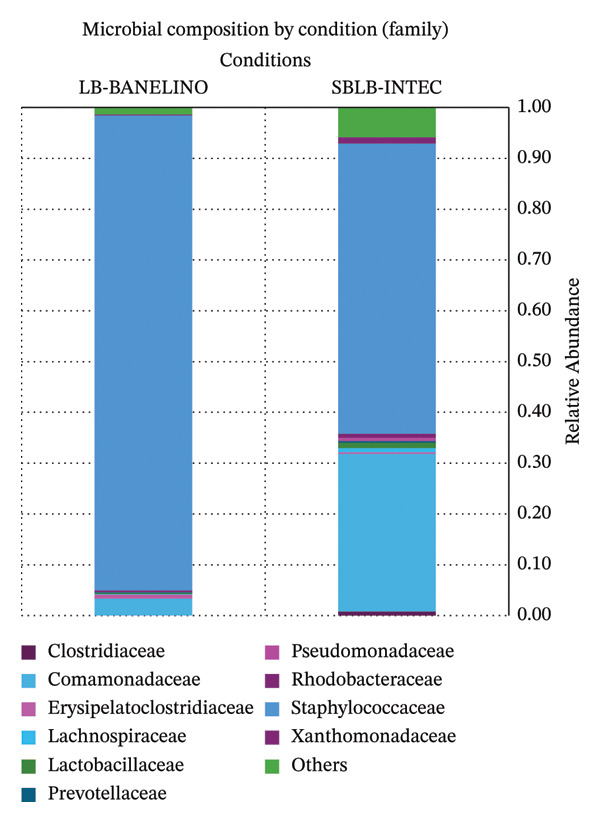
Relative abundance of bacterial families in SBLB‐INTEC and LB‐BANELINO.

Disaggregation by order and family shows that, in both formulations, the community is strongly enriched in taxa related to Staphylococcales/Staphylococcaceae, while Burkholderiales*/*Comamonadaceae are found almost exclusively in SBLB‐INTEC (Figures 3 and 4; Supporting Tables S6 and S7, relative abundance at the order and family levels, respectively).

Relative abundance (%) of the most abundant bacterial orders in each replicate of SBLB‐INTEC and LB‐BANELINO. The figure emphasizes the enrichment of Staphylococcales and the presence of Burkholderiales and other orders that differentiate the two formulations.

Stacked bar plots summarizing the relative abundance (%) of the most abundant bacterial families in each replicate. The figure highlights the dominance of Staphylococcaceae and the presence of Comamonadaceae and other families that contribute to differences between formulations.

**FIGURE 5:**
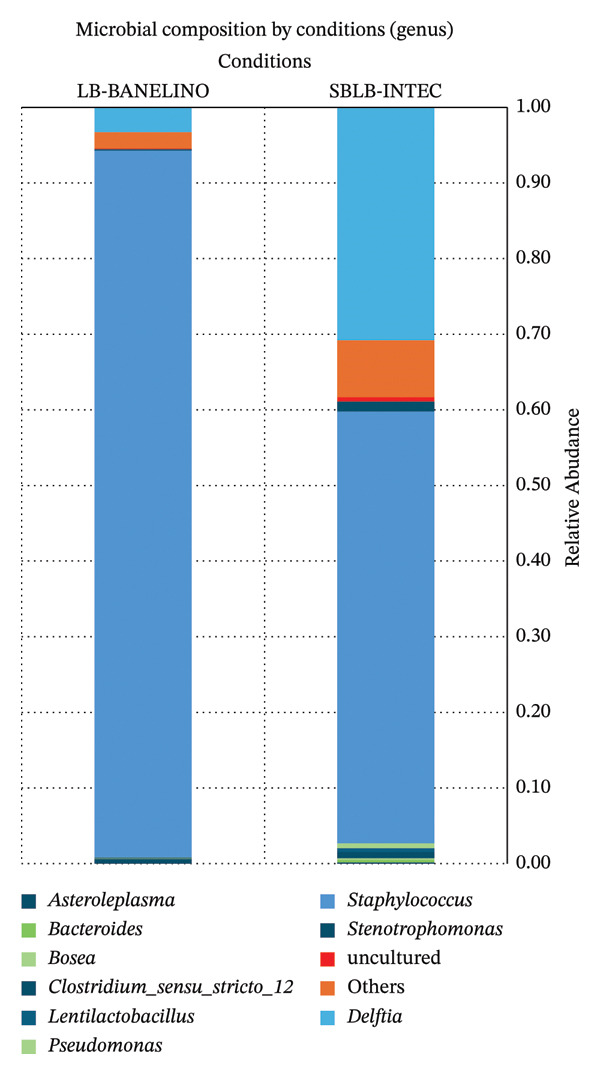
Relative abundance of bacterial genera in SBLB‐INTEC and LB‐BANELINO biofertilizers. Relative abundance (%) of the dominant bacterial genera in each replicate of SBLB‐INTEC and LB‐BANELINO. The plot shows the strong dominance of *Staphylococcus* across both products, with additional contributions from *Delftia* and other genera in SBLB‐INTEC.

At the genus level (Figure 5; Supporting Table S8), *Staphylococcus* dominates both formulations. In LB‐BANELINO, this genus accounts for, on average, more than 94% of the readings (range 94%–97%), whereas in SBLB‐INTEC, its relative abundance is lower but still very high (57%, range 55%–84%). In SBLB‐INTEC, a second dominant genus, *Delftia*, also appears, contributing around 30% of the bacterial community (range 11%–40%). Other genera, such as *Stenotrophomonas, Clostridium sensu stricto 12*, *Lentilactobacillus, Pseudomonas*, and *Prevotella*, are found in smaller proportions (< 5%).

These results are consistent with studies of commercial biofertilizers, where a few genera dominate the community, often belonging to the phyla Firmicutes and Proteobacteria [12]. Many members of the genera *Delftia*, *Stenotrophomonas*, and *Pseudomonas* have been described as plant growth‐promoting rhizobacteria, with the ability to solubilize phosphorus, produce siderophores, and modulate the availability of trace metals [49, 52]. However, these genera also include opportunistic strains, so it is crucial that we interpret the data with caution, underscoring the importance of your role in the research process.

The strong dominance of *Staphylococcus* in both biofertilizers is particularly relevant. On the one hand, various species of this genus have been isolated as plant *endophytes* or *epiphytes*, capable of producing secondary metabolites and contributing to nutrient recycling [47]. On the other hand, the genus includes opportunistic species of clinical and veterinary importance (*S. aureus, S. pseudintermedius*, among others). Analyses based on the V3–V4 region of 16S rRNA do not allow for robust discrimination between commensal, environmental, and pathogenic species within *Staphylococcus*. Therefore, although our data confirm that *Staphylococcus* is the dominant genus, it is crucial to recognize the limitations of 16S rRNA analysis for pathogenicity assessment and the need for further research and development in this area.

To explore the taxa of health interest in more detail, a subset of data were generated that includes reads assigned to *Staphylococcus* and *Escherichia–Shigella* (Supporting Tables S14–S17, which list raw counts, relative abundances, long‐format data, and presence/absence for genera that include potentially pathogenic species). In this subset, *Staphylococcus* constitutes more than 99.9% of the readings, while *Escherichia–Shigella* is detected only marginally. However, these tables should be interpreted solely as a description of genera that include potentially pathogenic species, not as confirmation of the presence of specific pathogens or their ability to cause disease.

#### 3.1.3. Agronomic Implications and Regional Context

From a biosecurity and functional perspective, the 16S rRNA amplicon data provide a high‐level description of the microbial communities present in SBLB‐INTEC and LB‐BANELINO. It is important to note that 16S rRNA sequencing has inherent methodological limitations: It cannot distinguish between viable and nonviable cells, cannot infer gene function or metabolic activity [50], and rarely resolves taxa at the species or strain level. Consequently, 16S rRNA profiles do not provide direct evidence of functional performance or microbiological safety.

The interpretations presented in this section are therefore derived from genus‐level taxonomic profiles and previously reported associations in the literature and should be regarded as ecological and agronomic context rather than demonstrated functional capabilities inferred directly from the sequencing data. Under these constraints, the detection of genera that may include opportunistic pathogens should be interpreted as an indication for targeted follow‐up analyses, rather than as direct evidence of health risk.

The principal contribution of the present dataset lies in its characterization of relatively dense yet structurally simple bacterial communities, dominated by Firmicutes, particularly Bacilli and lactic acid bacteria, with secondary contributions from Proteobacteria and other phyla. This community structure is consistent with the fermentative nature of both biofertilizer formulations and aligns with previous reports describing ecological roles of *Bacillus* and related Firmicutes in nutrient mobilization and plant‐associated systems [47, 48], as well as the emerging role of lactic acid bacteria in soil–plant interactions [53, 54].

SBLB‐INTEC showed slightly higher Shannon diversity indices, largely driven by contributions from Proteobacteria such as *Delftia* and other Comamonadaceae, suggesting a more even bacterial community in the *Sargassum*‐based formulation. Nevertheless, the absence of statistically significant differences in alpha diversity and the lack of clear separation in Bray–Curtis PCoA ordinations and PERMANOVA analyses indicate that both biofertilizers share a broadly similar overall community structure (Figure 6; Supporting Tables S9–S13).

FIGURE 6Alpha diversity and Bray–Curtis *β*‐diversity of bacterial communities in SBLB‐INTEC and LB‐BANELINO. (a) Boxplots of Shannon diversity indices and observed ASVs for SBLB‐INTEC (*n* = 3) and LB‐BANELINO (*n* = 3), calculated from the rarefied ASV table generated with the QIIME 2 core‐metrics‐phylogenetic pipeline. Individual data points for each replicate (SBLB_rep1–3, BANELINO_rep1–3) are shown overlaid on the boxplots. Per‐sample alpha diversity values are provided in Supporting Table S9, and summary statistics for between‐formulation comparisons (Mann–Whitney *U* tests) are reported in Supporting Table S10. (b) Principal coordinates analysis (PCoA) based on Bray–Curtis distances among samples, showing the ordination of the six bacterial communities colored by biofertilizer type. The underlying Bray–Curtis distance matrix and PCoA coordinates are provided in Supporting Tables S11 and S12, respectively. PERMANOVA results for differences in community composition between formulations are summarized in Supporting Table S13 and indicate no statistically significant separation between SBLB‐INTEC and LB‐BANELINO.

**Figure:**
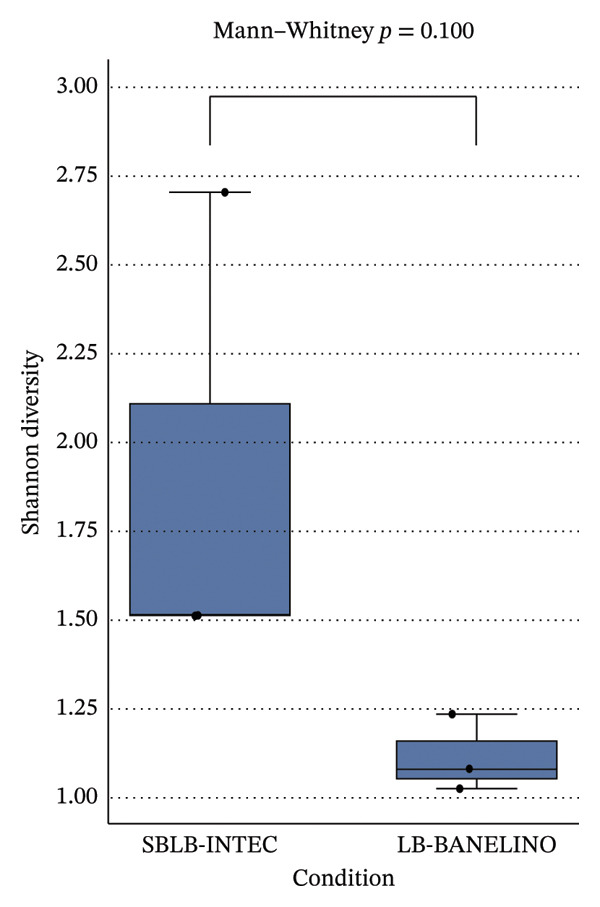
(a)

**Figure:**
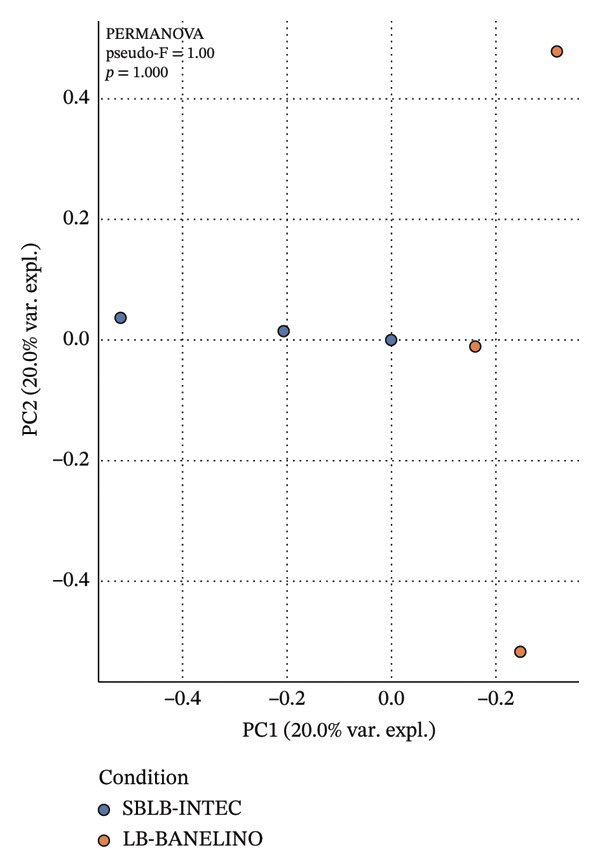
(b)

Phylogeny‐based beta‐diversity metrics were calculated using a rooted ASV phylogenetic tree constructed with MAFFT alignment and FastTree inference. This tree was used to compute Faith’s PD and UniFrac distance matrices, which incorporate evolutionary relationships among taxa rather than relying solely on taxon abundances. Inspection of UniFrac‐based ordinations indicated that samples tended to cluster according to biofertilizer formulation, while replicates within each formulation showed consistent phylogenetic patterns, indicating internal coherence.

Inspection of phylogeny‐based beta‐diversity patterns indicated that samples tended to cluster according to biofertilizer formulation, suggesting that differences between LB‐BANELINO and SBLB‐INTEC are associated not only with taxonomic composition but also with the distribution of phylogenetically related lineages. Replicates within each formulation showed consistent clustering, indicating internal phylogenetic coherence.

In the Dominican context, where export‐oriented banana systems and other staple crops depend heavily on imported mineral fertilizers, a locally produced liquid biofertilizer that valorizes massive *Sargassum* landings and agro‐industrial by‐products has clear strategic interest. Large *Sargassum* influxes along Caribbean coasts are an emerging environmental challenge, and their conversion into agricultural inputs is increasingly explored as part of circular bioeconomy approaches [3, 8]. The use of *Sargassum*‐based biofertilizers could help recycle nutrients, reduce reliance on synthetic fertilizers, and mitigate coastal pollution. These biofertilizers could also enhance soil health and crop productivity, thereby promoting sustainable and locally grounded agriculture in the Caribbean.

At the same time, the regional context urges a cautious, stepwise implementation. Recent work on Dominican river microbiomes has documented the circulation of bacteria with reduced susceptibility to beta‐lactam antibiotics in surface waters impacted by urban and industrial effluents [55]. Although our study did not evaluate antimicrobial resistance, this evidence highlights the importance of incorporating resistance monitoring into future assessments of residual‐origin biofertilizers.

Priority next steps for the Dominican Republic include deploying longitudinal studies of soil and rhizosphere microbiomes under repeated applications; conducting targeted surveys of pathogenic indicators and antibiotic‐resistance genes using qPCR and/or shotgun metagenomics; and conducting greenhouse and field trials to compare SBLB‐INTEC and LB‐BANELINO against sterile controls and conventional fertilization methods, with an emphasis on quantifying their effects on germination, biomass, and yield. This information will be crucial for assessing the safe and efficient application of liquid biofertilizers derived from *Sargassum* as part of the Caribbean’s transition to more locally based and sustainable agriculture.

## 4. Conclusions

This study provides the first detailed characterization, based on 16S rRNA ASVs, of the bacterial communities present in two liquid biofertilizers produced in the Dominican Republic. Both formulations are dominated by Firmicutes (class Bacilli) and the genus *Staphylococcus*. The dominance of these species could indicate their role in the efficacy or safety of biofertilizers. Additionally, there is an additional contribution from Gammaproteobacteria, especially *Delftia*, which is more pronounced in the SBLB‐INTEC. Alpha and beta diversity metrics showed no statistically significant differences between formulations, although a trend toward greater diversity was observed in SBLB‐INTEC.

The data obtained do not allow us to conclude that pathogens are absent or guarantee the microbiological safety of the products. This is due to the inherent limitations of 16S rRNA sequencing, which lacks negative controls or confirmatory tests. These limitations mean that the study’s findings are not definitive and should be used as a starting point for further, more targeted tests and field studies. Instead, the data provide a map of the genera present that can guide the design of targeted tests, such as qPCR, selective cultures, metagenomics, and field studies to evaluate both agronomic efficacy and health safety.

In the context of Dominican and Caribbean agriculture, these results underscore the promising potential of *Sargassum*‐based biofertilizers. They could play a significant role in sustainable nutrient management and coastal biomass valorization strategies. However, they also highlight the need for robust microbiological quality control frameworks that integrate genomic tools and functional assays before recommending their widespread use.

## Author Contributions

Conceptualization, Yaset Rodríguez‐Rodríguez, Edian F. Franco, Miguel Ángel Guevara, Rommel T. Ramos, and Ulises Javier Jáuregui‐Haza; methodology, Ashley Marie Mejía Disla, Máximo Elías Reynoso Ortega, Gustavo Gandini, and Pamela Tejada‐Tejada; software, Rommel T. Ramos, Edian F. Franco, and Carlos Willian Dias Dantas; validation, Ashley Marie Mejía Disla, Máximo Elías Reynoso Ortega, Gustavo Gandini, Carlos Willian Dias Dantas, and Pamela Tejada‐Tejada; formal analysis, Ashley Marie Mejía Disla, Yaset Rodríguez‐Rodríguez, Ulises Javier Jáuregui‐Haza, Edian F. Franco, Carlos Willian Dias Dantas and Pamela Tejada‐Tejada; investigation, Yaset Rodríguez‐Rodríguez, Ashley Marie Mejía Disla, Carlos Willian Dias Dantas and Pamela Tejada‐Tejada; resources, Yaset Rodríguez‐Rodríguez, Edian F. Franco, Miguel Ángel Guevara, Rommel T. Ramos, and Ulises Javier Jáuregui‐Haza; data curation, Rommel T. Ramos, Edian F. Franco, and Carlos Willian Dias Dantas; writing–original draft preparation, Yaset Rodríguez‐Rodríguez, Ashley Marie Mejía Disla, Máximo Elías Reynoso Ortega, Gustavo Gandini, and Pamela Tejada‐Tejada; writing–review and editing, Yaset Rodríguez‐Rodríguez, Edian F. Franco, Miguel Ángel Guevara, Rommel T. Ramos, and Ulises Javier Jáuregui‐Haza; visualization, Carlos Willian Dias Dantas; supervision, Yaset Rodríguez‐Rodríguez, Edian F. Franco, Miguel Ángel Guevara, Rommel T. Ramos, and Ulises Javier Jáuregui‐Haza; project administration, Yaset Rodríguez‐Rodríguez, Edian F. Franco, Miguel Ángel Guevara, Rommel T. Ramos, and Ulises Javier Jáuregui‐Haza; funding acquisition, Yaset Rodríguez‐Rodríguez, Edian F. Franco, Miguel Ángel Guevara, Rommel T. Ramos, and Ulises Javier Jáuregui‐Haza.

## Funding

This research was funded by Ministerio de Educación Superior, Ciencia y Tecnología (MESCYT) through Fondo Nacional de Innovación y Desarrollo Científico y Tecnológico (FONDOCYT), grant number 2020‐2021‐2C6‐029: Abordaje de OneHealth para la mejora de la calidad e inocuidad de los vegetales y hortalizas producidos y comercializados en República Dominicana a través de las ciencias ómicas y bioinformática.

## Disclosure

All authors have read and agreed to the published version of the manuscript.

## Conflicts of Interest

The authors declare no conflicts of interest.

## Supporting Information

Supporting Table S1: ASV count matrix of all samples after quality filtering and denoising.

Supporting Table S2: Relative abundance of bacterial taxa across all samples.

Supporting Table S3: Taxonomic classification of ASVs based on the SILVA 138 database.

Supporting Table S4A: Sample metadata used for bioinformatic and statistical analyses.

Supporting Table S5A: Sequencing read counts at each bioinformatic processing step.

Supporting Table S6A: Sequencing quality metrics for each sample after quality filtering and chimera removal.

Supporting Table S4: Relative abundance of phyla by sample.

Supporting Table S5: Relative abundance of bacterial classes by sample.

Supporting Table S6: Relative abundance of data order by sample.

Supporting Table S7: Relative abundance of data family by sample.

Supporting Table S8: Relative abundance of data for the main genera by sample.

Supporting Tables S9–S13: Alpha and beta diversity metrics and associated statistical analyses, including Bray–Curtis distances, PCoA, and PERMANOVA results.

Supporting Tables S14–S17: Genera including potentially pathogenic taxa, including raw counts, relative abundance, and presence/absence data.

Supporting Figure S1A: Rarefaction curves based on observed ASVs and Shannon diversity.

## Supporting information


**Supporting Information** Additional supporting information can be found online in the Supporting Information section.

## Data Availability

Raw sequencing data are available under NCBI BioProject ID PRJNA1237349.
