# Supplementary material for: Microbial Profiling and Biosafety Assessment of a Sargassum-Based Liquid Biofertilizer Using 16S rRNA Metagenomics
Source: Int J Microbiol. 2026 Apr 24;2026:3219583. doi: 10.1155/ijm/3219583 (PMC13107165; doi:10.1155/ijm/3219583)
Supplement: Supplementary file 1 — Supporting Information Additional supporting information can be found online in the Supporting Information section. [file IJM-2026-3219583-s001.zip › Supplementary Figure S1A.docx]

Supplementary Figure S1A


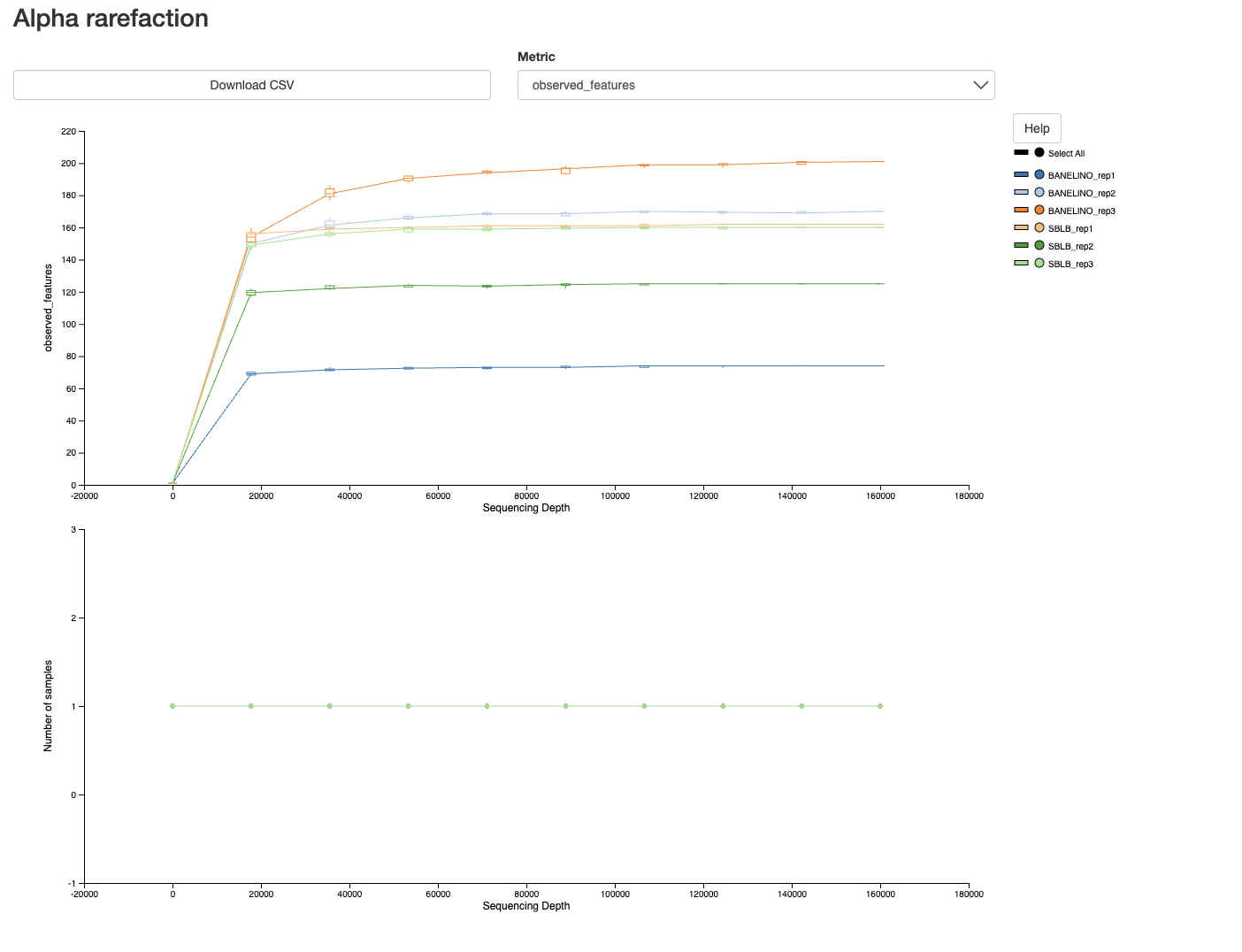


**Supplementary Figure S1A.** Alpha rarefaction curves based on observed amplicon sequence variants (ASVs) for all biofertilizer samples. Curves show saturation at moderate sequencing depths, supporting adequate coverage of bacterial richness in all samples.


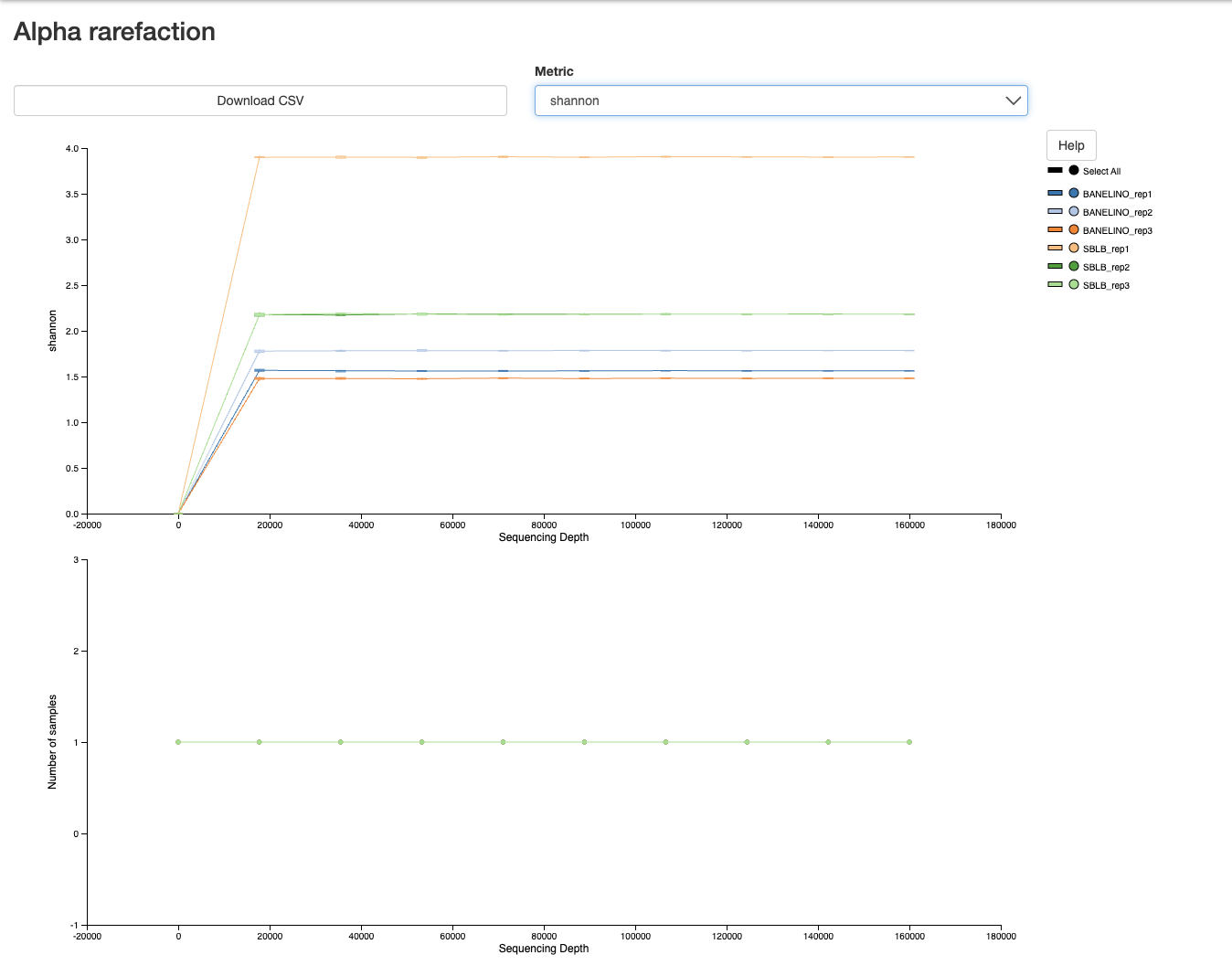


**Supplementary Figure S1B.** Alpha rarefaction curves based on the Shannon diversity index for all biofertilizer samples. Shannon diversity values stabilize at low sequencing depths, indicating that community structure is not affected by sequencing effort.
